# Cyclin D1 in ASM Cells from Asthmatics Is Insensitive to Corticosteroid Inhibition

**DOI:** 10.1155/2012/307838

**Published:** 2012-02-19

**Authors:** Jodi C. Allen, Petra Seidel, Tobias Schlosser, Emma E. Ramsay, Qi Ge, Alaina J. Ammit

**Affiliations:** ^1^Respiratory Research Group, Faculty of Pharmacy, University of Sydney, Sydney, NSW 2006, Australia; ^2^Woolcock Institute of Medical Research, University of Sydney, Sydney, NSW 2006, Australia

## Abstract

Hyperplasia of airway smooth muscle (ASM) is a feature of the remodelled airway in asthmatics. We examined the antiproliferative effectiveness of the corticosteroid dexamethasone on expression of the key regulator of G_1_ cell cycle progression—cyclin D1—in ASM cells from nonasthmatics and asthmatics stimulated with the mitogen platelet-derived growth factor BB. While cyclin D1 mRNA and protein expression were repressed in cells from nonasthmatics in contrast, cyclin D1 expression in asthmatics was resistant to inhibition by dexamethasone. This was independent of a repressive effect on glucocorticoid receptor translocation. Our results corroborate evidence demonstrating that corticosteroids inhibit mitogen-induced proliferation only in ASM cells from subjects without asthma and suggest that there are corticosteroid-insensitive proliferative pathways in asthmatics.

## 1. Introduction

Asthma is a chronic inflammatory condition of the lung associated with structural remodelling of the airway wall. As a consequence of long-term exposure to inflammatory mediators, the airways of asthmatics become remodelled. Airway smooth muscle mass is increased, and neovascularization is evident in the subepithelial mucosa. Airway fibrosis becomes apparent, with thickening of the lamina reticularis and increased interstitial extracellular matrix deposition being typical features of an asthmatic airway [1]. Although numerous cell types contribute to airway remodelling, the increase in airway smooth muscle mass is considered to have the largest impact on airway narrowing in asthma [2–4].

Inhaled corticosteroids are a first-line anti-inflammatory therapy in asthma. However, as many asthmatics manifest persistent airway hyperresponsiveness even after prolonged corticosteroid therapy [5], corticosteroid resistance and insensitivity is known to exist [6]. Although corticosteroids can inhibit some aspects of remodelling [7], we do not yet know whether ASM mass is reduced by corticosteroid treatment *in vivo*. Interestingly, Roth et al. [8] demonstrated that ASM proliferation in cells derived from asthmatics is resistant to inhibition by corticosteroids, suggesting that the proliferative pathways underlying the hyperplastic ASM phenotype in asthmatics are corticosteroid insensitive. 

In this study, we continue investigations into corticosteroid insensitivity *in vitro* by examining the effect of dexamethasone on a key regulator of G_1_ cell cycle progression, cyclin D1, in ASM cells from asthmatics and nonasthmatics. Cyclin D1 has been the most widely studied cyclin in ASM biology using cells from nonasthmatics [4, 9, 10] and more recently asthmatics [11]. Our study examines cyclin D1 mRNA and protein expression in ASM cells from asthmatics and demonstrates that cyclin D1 upregulation is insensitive to corticosteroid inhibition. 

## 2. Materials and Methods 

### 2.1. Cell Culture

Human ASM cells were obtained from subjects without and with asthma by methods adapted from those previously described [12, 13], in accordance with procedures approved by the Sydney South West Area Health Service and the Human Research Ethics Committee of the University of Sydney. A minimum of three different ASM primary cell lines were used for each experiment. All the subjects' disease states were confirmed by doctor diagnosis, and subject demographics are shown in Table 1. 

Unless otherwise specified, all chemicals used in this study were purchased from Sigma-Aldrich (St. Louis, MO). 

### 2.2. Cyclin D1 mRNA Expression

To examine the time course of induction of cyclin D1 mRNA expression by platelet-derived growth factor BB (PDGF-BB) and repression by dexamethasone, growth-arrested ASM cells from *n* = 8 nonasthmatic and *n* = 7 asthmatic subjects were pretreated for 1 h with 100 nM dexamethasone, compared to vehicle. Cells were then stimulated with PDGF-BB (25 ng/mL: Merck, Darmstadt, Germany) for 0, 2, 4, 8, and 24 h, and cyclin D1 mRNA expression was quantified by real-time RT-PCR as previously described [14].

### 2.3. Cyclin D1 Protein Expression

To examine the time course of cyclin D1 protein upregulation by PDGF-BB and repression by dexamethasone, growth-arrested ASM cells from *n* = 7 non-asthmatic and *n* = 7 asthmatic subjects were pretreated for 1 h with 100 nM dexamethasone, compared to vehicle. Cells were then stimulated with 25 ng/mL PDGF-BB for 0, 2, 4, 8, and 24 h. Cells were lysed, then cyclin D1 was quantified by western blotting using a rabbit polyclonal antibody against cyclin D1 (M-20: Santa Cruz Biotechnology, Santa Cruz, CA), compared to *α*-tubulin as the loading control (mouse monoclonal IgG_1_, clone DM 1A; Santa Cruz). Primary antibodies were detected with goat anti-mouse or anti-rabbit horse-radish peroxidase-conjugated secondary antibodies (Cell Signaling Technology, Danvers, MA) and visualized by enhanced chemiluminescence (PerkinElmer, Wellesley, MA). Densitometry was performed using Image J [15].

### 2.4. Translocation

To measure translocation of the glucocorticoid receptor (GR), growth-arrested ASM cells from *n* = 3 non-asthmatic and *n* = 3 asthmatic subjects were treated with vehicle or dexamethasone (100 nM) for 1 h, prior to stimulation with 25 ng/mL PDGF-BB for 1 h. Cytoplasmic and nuclear protein extraction was performed using NE-PER nuclear and cytosolic extraction kit according to the manufacturer's instructions (Thermo Fisher Scientific, Rockford, IL). GR was quantified by western blotting using a rabbit polyclonal antibody against GR (E-20: Santa Cruz Biotechnology) compared to *α*-tubulin and a rabbit polyclonal antibody to lamin A/C (cell signaling technology) as a loading control for the cytosolic and nuclear fractions, respectively.

### 2.5. Statistical Analysis

Statistical analysis was performed using either the Student's unpaired *t-*test, or one-way or two-way ANOVA followed by Fisher's post hoc multiple comparison test. *P* values <0.05 were sufficient to reject the null hypothesis for all analyses. 

## 3. Results

**Figure 1:**
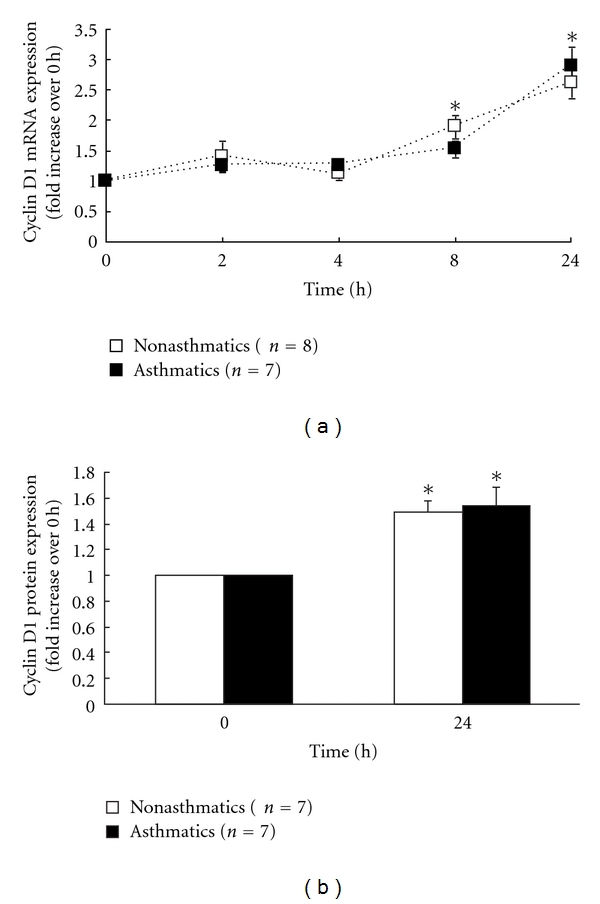
PDGF-BB upregulates cyclin D1 mRNA and protein expression in ASM from nonasthmatics and asthmatics. ASM cells from non-asthmatics and asthmatics were stimulated with 25 ng/mL of mitogen PDGF-BB. (a) demonstrates the temporal kinetics of cyclin D1 mRNA expression quantified by real-time RT-PCR (expressed as fold increase over 0 h). Statistical analysis was performed using one-way ANOVA, followed by Fisher's post hoc multiple comparison test (∗ denotes a significant effect of PDGF-BB on cyclin D1 mRNA, compared to 0 h (*P* < 0.05)). (b) shows densitometric analysis of cyclin D1 protein expression at 24 h quantified by western blotting (expressed as fold increase over 0 h), using *α*-tubulin as the loading control. Statistical analysis was performed using the Student's unpaired *t-*test (where ∗ denotes a significant effect of PDGF-BB on cyclin D1 protein, compared to 0 h (*P* < 0.05)). Values are mean + SE.

To examine the time course of induction of cyclin D1 mRNA by the mitogen PDGF-BB, growth-arrested ASM cells from non-asthmatic and asthmatic subjects were stimulated with PDGF-BB for up to 24 h. As shown in Figure 1(a), a significant increase in cyclin D1 mRNA expression was first detected 8 h after PDGF-BB treatment. By 24 h, cyclin D1 mRNA expression had further significantly increased to 2.6 ± 0.3-fold in ASM cells from non-asthmatics and 2.9 ± 0.3-fold in cells from asthmatics (*P* < 0.05). Interestingly, there was no significant difference between the increases in cyclin D1 upregulation in cells from asthmatics, as compared to nonasthmatic controls. Cyclin D1 protein expression at 24 h was similarly upregulated in support of the mRNA data Figure 1(b). Interestingly, there were no significant differences between the amount of cyclin D1 mRNA and protein expression in the asthmatics, as compared to non-asthmatics. 

**Figure 2:**
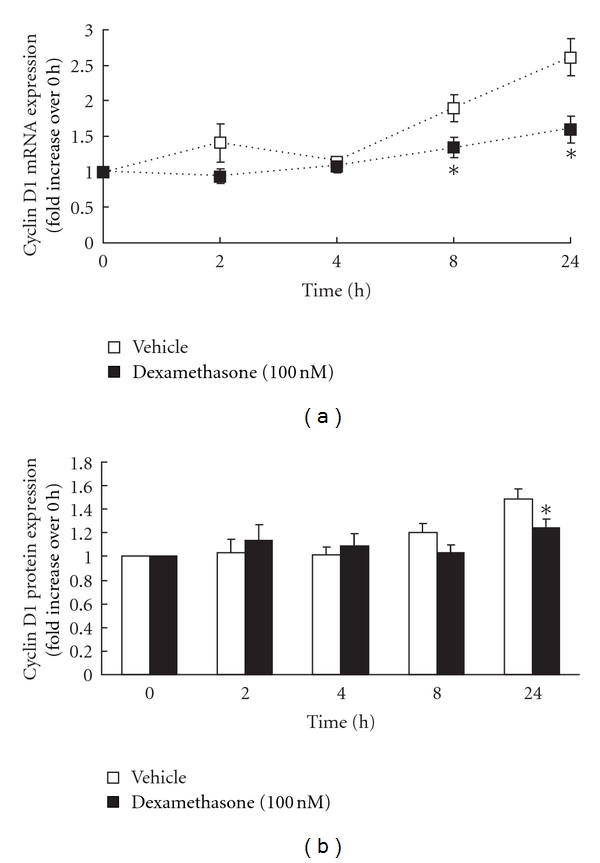
Dexamethasone inhibits cyclin D1 mRNA and protein expression in ASM cells from non-asthmatics. ASM cells from non-asthmatics (*n* = 7 − 8) were pretreated for 1 h with vehicle or dexamethasone and then stimulated with 25 ng/mL PDGF-BB. (a) demonstrates the effect of dexamethasone on the temporal kinetics of PDGF-BB-induced cyclin D1 mRNA expression (expressed as fold increase over 0 h). Statistical analysis was performed using two-way ANOVA, followed by Fisher's post hoc multiple comparison test (where ∗ denotes significant inhibition by dexamethasone (*P* < 0.05)). (b) shows densitometric analysis of the effect of dexamethasone on cyclin D1 protein (expressed as fold increase over 0 h), using *α*-tubulin as the loading control. Statistical analysis was performed using the Student's unpaired *t*-test (where ∗ denotes a significant inhibition by dexamethasone (*P* < 0.05)). Values are mean + SE.

We then examined the effect of the corticosteroid dexamethasone on the temporal kinetics of PDGF-BB-induced cyclin D1 mRNA and protein expression in ASM cells from asthmatics and non-asthmatics. Growth-arrested ASM cells were pretreated for 1 h with vehicle or 100 nM dexamethasone and stimulated with PDGF-BB for up to 24 h. In ASM cells from non-asthmatics, the corticosteroid dexamethasone significantly inhibited cyclin D1 mRNA upregulation in response to PDGF-BB stimulation (Figure 2(a), *P* < 0.05). As shown in Figure 2(a), 100 nM dexamethasone significantly inhibited the amount of PDGF-BB-induced cyclin D1 mRNA expression at 8 h and 24 h after stimulation (Figure 2(a); *P* < 0.05), and a resultant attenuation of cyclin D1 protein expression was also observed at 24 h (Figure 2(b); *P* < 0.05). 

**Figure 3:**
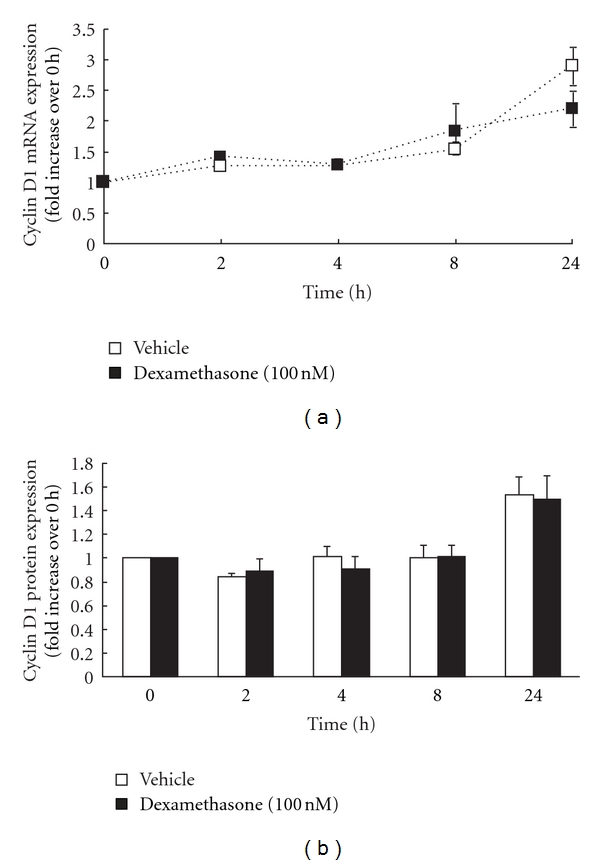
Cyclin D1 mRNA and protein expression in ASM cells from asthmatics were relatively resistant to inhibition by dexamethasone. ASM cells from asthmatics (*n* = 7) were pretreated for 1 h with vehicle or dexamethasone and then stimulated with 25 ng/mL PDGF-BB. (a) demonstrates the effect of dexamethasone on the temporal kinetics of PDGF-BB-induced cyclin D1 mRNA expression (expressed as fold increase over 0 h). (b) shows densitometric analysis of the effect of dexamethasone on cyclin D1 protein (expressed as fold increase over  h), using *α*-tubulin as the loading control. Values are mean +  SE.

In contrast, parallel experiments performed in ASM cells from asthmatics revealed that the PDGF-BB-induced upregulation of cyclin D1 mRNA and protein expression was resistant to inhibition by dexamethasone (Figure 3). As shown in Figure 3(a), there was no significant difference in the temporal kinetics of PDGF-BB-induced cyclin D1 mRNA expression in the presence or absence of dexamethasone. Moreover, there were no significant differences in amounts of PDGF-BB-induced cyclin D1 protein induced after pretreatment with dexamethasone (1.5 ± 0.2-fold), as compared to vehicle control (1.5 ± 0.2-fold) (Figure 3(b)).

**Figure 4:**
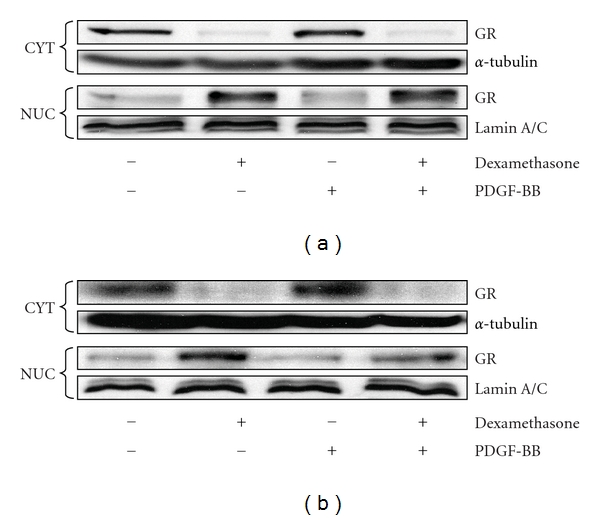
Dexamethasone-induced GR nuclear translocation does not differ between mitogen-treated ASM from asthmatics as compared to non-asthmatics. ASM cells from (a) *n* = 3 non-asthmatics or (b) *n* = 3 asthmatics were pretreated for 1 h with vehicle or dexamethasone (100 nM) and then stimulated for 1 h with 25 ng/mL PDGF-BB. Cytoplasmic (CYT) and nuclear (NUC) fractions were prepared, and GR protein was measured by western blotting (representative blots illustrated), using *α*-tubulin and lamin A/C as the loading controls for the cytoplasmic or nuclear fractions, respectively.

To examine whether the degree of GR nuclear translocation differed in mitogen-treated ASM from asthmatics as compared to non-asthmatics, cells were pretreated with dexamethasone for 1 h, stimulated with PDGF-BB for 1 h, prior to preparation of purified nuclear and cytoplasmic extracts. As shown in Figure 4, dexamethasone induced the translocation of GR to a similar extent in cells from non-asthmatics (Figure 4(a)), compared to asthmatics (Figure 4(b)). PDGF-BB had no effect on the degree of GR nuclear translocation induced by dexamethasone. 

## 4. Discussion

Increase in airway smooth muscle mass is a hallmark of the remodelled airway in asthma. Many studies have focused on the G_1_-to-S transition, in particular the role of cyclin D1 in modulating G_1_ progression to S-phase traversal in ASM cells from non-asthmatics. Our study examines cyclin D1 in ASM from asthmatic subjects, and compares the relative inhibitory efficacy of corticosteroids with ASM cells from non-asthmatics. We have confirmed that cyclin D1 upregulation in ASM cells is inhibited by corticosteroids [16]. We are the first to examine the effect of dexamethasone on the mitogen PDGF-BB-induced cyclin D1 upregulation in ASM cells from both non-asthmatics and asthmatics. Intriguingly, we show that we are unable to totally inhibit cyclin D1 mRNA and protein upregulation in cells from asthmatic subjects. These results corroborate earlier evidence demonstrating that corticosteroid-inhibited proliferation occurs only in ASM cells from subjects without asthma [8] and suggest that there are corticosteroid insensitive proliferative pathways in asthmatics that warrant further investigation in order to design efficacious antiremodelling strategies.

How ASM mass increases in a remodelled airway is an area under intense investigation. *In vivo*, there is evidence of greater ASM cell number (hyperplasia) [17, 18]*. In vitro*, hyperplasia of ASM is well supported by numerous *in vitro *studies in which ASMs have been shown to proliferate in response to mitogenic inflammatory mediators present in the inflamed airways (reviewed in [4, 9, 10]), and by the observation that ASM cells from asthmatics have a greater rate of proliferation *in vitro* when compared to cells from non-asthmatics [13, 19]. Cyclin D1 upregulation, and its repression by corticosteroids, has been extensively examined in *in vitro* studies using cells from non-asthmatics (reviewed in [4, 9, 10]). Our study compares and contrasts the upregulation of cyclin D1, and the relative efficacy of corticosteroids, in cells from both asthmatics and non-asthmatics examined *in vitro*. We observe corticosteroid insensitivity in cells from asthmatics. This insensitivity was not due to altered nuclear translocation of the glucocorticoid receptor in ASM cells from asthmatics compared to non-asthmatics. Rather, a number of studies may have posed some possible explanations for our observations. ASM cells are tethered within the extracellular-matrix- (ECM-) rich microenvironment of fibrotic asthmatic airway. In asthma, the abundance of ECM proteins, including collagen I, is increased [20]. Bonaccci et al. [21] demonstrated that integrin/collagen I interactions impaired the antimitogenic action of dexamethasone. As ASM cells from asthmatics secrete greater amounts of collagen I than non-asthmatics, integrin/ECM interactions may contribute to corticosteroid resistance in our experiments. As an alternative explanation, Roth et al. [8] demonstrated that the antiproliferative effect of corticosteroids in ASM cells requires the formation of a complex between the glucocorticoid receptor and the CCAAT/enhancer binding protein alpha (C/EBP*α*). As C/EBP*α* protein is absent in ASM cells from asthmatics, interaction of corticosteroid with its cognate receptor would be unable to form the required anti-mitogenic complex, explaining the failure of corticosteroids to inhibit cyclin D1-mediated proliferative pathways *in vitro*. 

In summary, we have shown that cyclin D1 mRNA and protein upregulation in asthmatic ASM cells are insensitive to inhibition by corticosteroids. Our results may reflect the contribution of impaired corticosteroid action via ASM-ECM interactions or absence of a key transcriptional regulator of corticosteroid action (C/EBP*α*) in asthmatic cells. Which possibility is responsible for corticosteroid insensitivity in ASM cells from asthmatics is unknown at present, but this observation underscores the importance of investigations into underlying molecular mechanisms of ASM hyperplasia in asthma to allow future development of efficacious antiremodelling strategies as future asthma pharmacotherapeutics.

## Figures and Tables

**Table 1:** Subject demographics.

Subject no.	Gender	Disease	FEV1 (% Pred)	FVC (% Pred)	FEV1 : FVC (%)	Smoker	Height	Weight	Experiments
1	F	Ca							1, 2
2	F	A	64%	70%	76%	Ex-smoker			1, 3
3	F	Ca							1, 2
4	M	A	82%			No			1, 3
5	F	NSCCa				Yes			1, 2
6	F	A	2.04 L	2.5 L		No	158 cm	64 kg	1, 3
7	M	NSSCa	2.12 L	3.35 L		Ex-smoker	173 cm	76 kg	1, 2
8	F	A	1.87 L	2.32 L		No	168 cm	57 kg	1, 3
9	F	Bronchiolitis obliterans							1, 2
10	M	A	3.75 L	5.9 L		No	179 cm	110 kg	1, 3
11	M	Emphysema							1, 2
12	M	A				No			1, 3
13	F	Emphysema				Yes			1, 2
14	M	A	4.77 L	5.32 L		Yes			1, 3
15	F	Emphysema	27% (0.51 L)	67% (1.5 L)			153 cm		1, 2
16	M	Emphysema, *α*-1 antitrypsin deficiency	15% (0.61 L)	66% (3.42 L)					4
17	M	A	57%	61%	77%	No			4
18	M	COPD	20%	65%	24%	Yes			4
19	M	Ca + A				Ex-smoker			4
20	M	Ca	1.95 L	2.5 L		Ex-smoker	169 cm	79 kg	4
21	M	A							4

Abbreviations used: Ca, carcinoma; A, asthma; NSCCa, nonsmall cell carcinoma; COPD, chronic obstructive pulmonary disease; FEV1, forced expiratory volume in 1 second (% predicted); FVC, forced vital capacity (% predicted).
